# Histology-based survival outcomes in hormone receptor-positive metastatic breast cancer treated with targeted therapies

**DOI:** 10.1038/s41523-022-00499-7

**Published:** 2022-12-20

**Authors:** Jason A. Mouabbi, Akshara Singareeka Raghavendra, Roland L. Bassett, Amy Hassan, Debu Tripathy, Rachel M. Layman

**Affiliations:** 1 Department of Breast Medical Oncology, Unit 1354, The University of Texas MD Anderson Cancer Center, 1515 Holcombe Blvd., Houston, TX 77030 USA; 2 Department of Biostatistics, Unit 1411, The University of Texas MD Anderson Cancer Center, 1515 Holcombe Blvd., Houston, TX 77030 USA; 3 Department of General Oncology, Unit 462, The University of Texas MD Anderson Cancer Center, 1515 Holcombe Blvd., Houston, TX 77030 USA

**Keywords:** Breast cancer, Breast cancer

## Abstract

The addition of targeted therapies (TT) to endocrine therapy (ET) has improved the outcomes of patients with HR-positive, HER2-negative metastatic breast cancer (mBC). However, it is unknown whether patients with invasive lobular carcinoma (ILC) or mixed invasive ductal and lobular carcinoma (mixed) histologies experience the same magnitude of benefit from this therapy as those with invasive ductal carcinoma (IDC). We aim to determine whether patients with IDC, ILC, and mixed HR+/HER2− mBC derive similar benefit from the addition of cyclin-dependent kinase 4 and 6 inhibitors (CDK4/6is), mammalian target of rapamycin inhibitor (mTORi), and phosphoinositide 3-kinase inhibitor (PI3Ki) to ET in HR+/HER2− mBC. We conducted an observational, population-based investigation using data from the MD Anderson prospectively collected database. We conducted a histology-based analysis of progression-free survival (PFS) and overall survival (OS) durations in 3784 patients with HR+/HER2− mBC who were treated with TT plus ET between January 1, 2010, and December 31, 2021. Out of the 3784 patients, 2975 were included in the final analysis. Of these, 2249 received CDK4/6is (81% IDC, 15% ILC, and 4% mixed), 1027 received everolimus (82% IDC, 14% ILC, and 4% mixed) and 49 received alpelisib (81% IDC and 19% ILC). The addition of targeted therapy to ET did not result in statistically significant differences in PFS or OS duration among patients with IDC, ILC, and mixed HR+/HER2− mBC. We concluded that for patients with HR+/HER2− mBC, the addition of TT to ET leads to a similar magnitude of benefit, irrespective of histology.

## Introduction

Invasive breast cancer (BC) is composed of more than 20 different histological subtypes. The most common is invasive ductal carcinoma (IDC), also commonly classified as invasive carcinoma of no special type, which accounts for 80% of all invasive BCs^1^, followed by invasive lobular carcinoma (ILC) (10–15%)^2^ and mixed invasive ductal and lobular carcinoma (mixed), which is often misclassified as ILC (5%)^3^.

ILC is distinct from IDC in its clinicopathologic characteristics and molecular alterations^4,5^. One special feature of ILC is the near-universal loss of the cell adhesion protein E-cadherin in ~90% of cases^6^) because of a loss of function via genomic loss (most commonly heterogenous 16q [90–94% of cases]^6–9^) or mutation^4^. ILC generally has features that are associated with a good prognosis, most often a low grade, low proliferation index, and strong ER positivity^10^. However, compared to IDC, ILC tends to have a higher risk of distant recurrence after 10 years^11^ and tends to exhibit peculiar metastatic patterns^12^. ILC also differs in its response to systemic therapy^13^, responding more poorly to chemotherapy than IDC^14^. Furthermore, ILC may exhibit partial intrinsic resistance to tamoxifen, a hypothesis supported by cell line studies^15^.

The majority (93%) of metastatic ILCs are hormone receptor-positive and human epidermal growth factor receptor 2-negative (HR+/HER2−)^11^. Endocrine therapy (ET) is recommended for HR+/HER2− metastatic BC (mBC), but its effectiveness as a single agent is limited by high rates of de novo and acquired resistance. Only about 30% of patients with metastatic ILC experience objective regression of their tumor with initial ET, and another 20% have prolonged stable disease^16^. Numerous escape pathways to ER targeting have been described that may be active at treatment initiation or evolve over the course of therapy^17^.

Understanding the mechanisms of ET resistance has informed the development of targeted therapies^18^. One such mechanism is the role of cell cycle signaling pathways in both oncogenesis and anti-estrogen resistance, which led to the development of cyclin-dependent kinase 4/6 inhibitors (CDK4/6is) that transform the management of HR+/HER2− mBC^19^. Another important mechanism is the alteration of the phosphatidylinositol 3-kinase (PI3K)/AKT/mammalian target of the rapamycin (mTOR) pathway, which is known to be vital in mBC cell growth and drives ET resistance. Drugs targeting PI3K and mTOR are currently used in clinical practice in patients who experience disease progression on CDK4/6is plus ET^20^.

In most BC clinical trials, enrollment criteria do not discriminate between histology; thus, they are not powered to detect histology-based differences in outcomes. It is currently unknown whether patients with ILC or mixed histology derive similar benefits from treatment with ET in combination with CDK4/6is, mTORi, or PI3Ki as do those with IDC. Therefore, we determined whether patients with IDC, ILC, and mixed HR+/HER2− mBC derive similar benefit from the addition of CDK4/6is, mTORi, and PI3Ki to ET in a retrospective observational, population-based investigation.

## Results

### Baseline characteristics

**Fig. 1:**
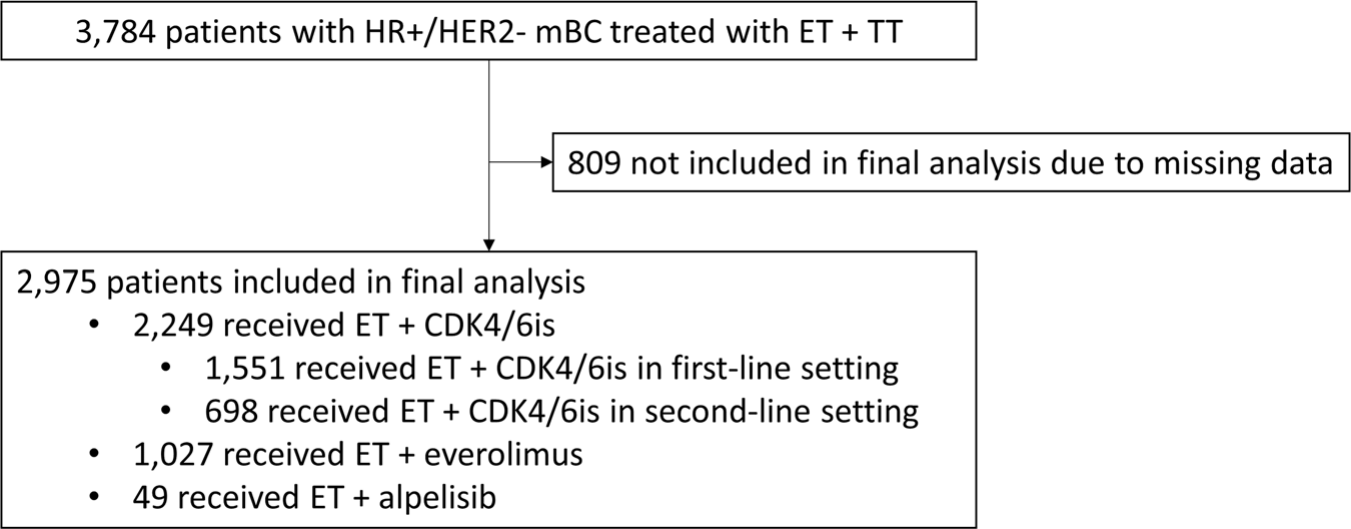
Consort diagram. ET endocrine therapy, TT targeted therapy, CDK4/6is cyclin-dependent kinase 4 and 6 inhibitors.

Between 2010 and 2021, we identified 3784 HR + /HER2− mBC patients who were treated with ET + TT at MD Anderson and who were included in the BC database. Of these, 2975 patients were included in the final analysis (809 had missing data and could not be included) (Fig. 1): 2249 (81% IDC, 15% ILC, and 4% mixed) received CDK4/6is in combination with ET, 1027 (82% IDC, 14% ILC, and 4% mixed) received the mTORi everolimus in combination with ET, and 49 (81% IDC and 19% ILC) received the PI3Ki alpelisib in combination with ET (Table 1). The median follow-up time for the study population is 18.8 months.

**Table 1:** Patient characteristics.

Characteristic	CDK4/6is + ET, *n* = 2249			Everolimus + ET, *n* = 1027			Alpelisib + ET, *n* = 49	
	IDC, *n* = 1834 (81%)	ILC, *n* = 336 (15%)	Mixed, *n* = 79 (4%)	IDC, *n* = 843 (82%)	ILC, *n* = 141 (14%)	Mixed, *n* = 43 (4%)	IDC, *n* = 40 (82%)	ILC, *n* = 9 (18%)
Age (years), median	49	54	57	48	53	48	51	52
Race, *n* (%)								
White	1375 (75)	269 (80)	59 (75)	613 (73)	114 (81)	35 (81)	37 (93)	7 (78)
Hispanic	147 (8)	34 (10)	10 (13)	87 (10)	13 (9)	5 (12)	1 (3)	1 (11)
Black	165 (9)	17 (5)	5 (6)	82 (10)	5 (4)	2 (5)	2 (5)	0
Others	147 (8)	16 (5)	5 (6)	63 (8)	9 (6)	1 (2)	0	1 (11)
ILC subtype, *n* (%)								
Classic		318 (95)			130 (92)			9 (100)
Pleomorphic		18 (5)			11 (8)			0
Menopausal status, *n* (%)								
Pre	1064 (58)	148 (44)	27 (35)	464 (55)	59 (42)	23 (54)	19 (48)	4 (44)
Post	770 (42)	188 (56)	52 (66)	379 (45)	82 (58)	20 (47)	21 (53)	5 (56)
Estrogen receptor, *n* (%)								
Positive	1779 (97)	333 (99)	77 (98)	778 (92)	138 (98)	42 (98)	37 (93)	9 (100)
Negative	55 (3)	3 (1)	2 (3)	64 (8)	3 (2)	1 (2)	3 (8)	0
Progesterone receptor, *n* (%)								
Positive	1577 (86)	285 (85)	67 (85)	655 (78)	120 (85)	37 (86)	35 (88)	9 (100)
Negative	257 (14)	51 (15)	9 (11)	187 (22)	20 (14)	6 (14)	5 (13)	0

*CDK4/6is* cyclin-dependent kinase 4 and 6 inhibitors, *ET* endocrine therapy, *IDC* invasive ductal carcinoma, *ILC* invasive lobular carcinoma, mixed, mixed invasive ductal and lobular carcinoma.

Others include Asian and Native American.

The median age was 51 years in all groups, with 54% of patients being postmenopausal. Most (75%) patients were non-Hispanic White, and 9% were Hispanic (Table 1). Among the patients who received CDK4/6is plus ET, 93% (all histological types) received palbociclib; around 70% received CDK4/6is in the 1L setting, and around 65% of the ET backbone in 1L was an AI (Table 2). All the patients who received FUL in the 1L setting experienced disease recurrence while on adjuvant AI.

**Table 2:** Characteristics of patients treated with CDK4/6is plus ET.

Treatment characteristic	CDK4/6is + ET, *n* = 2249		
	IDC, *n* = 1834 (81%)	ILC, *n* = 336 (15%)	Mixed, *n* = 79 (4%)
CDK4/6is, *n* (%)			
Palbociclib	1709 (93)	310 (92)	74 (94)
Ribociclib	51 (3)	5 (2)	2 (3)
Abemaciclib	74 (4)	21 (6)	3 (4)
Line of therapy, *n* (%)			
1L	1245 (68)	248 (74)	58 (73)
2L+	589 (32)	88 (26)	21 (27)
ET backbone in 1L, *n* (%)			
Aromatase inhibitor	829 (67)	158 (64)	34 (59)
Fulvestrant	416 (33)	90 (36)	24 (41)

*CDK4/6is* cyclin-dependent kinase 4 and 6 inhibitors, *ET* endocrine therapy, *IDC* invasive ductal carcinoma, *ILC* invasive lobular carcinoma, mixed mixed invasive ductal and lobular carcinoma, *1L* first line, *2L+* second line and beyond.

### Treatment outcomes

**Fig. 2:**
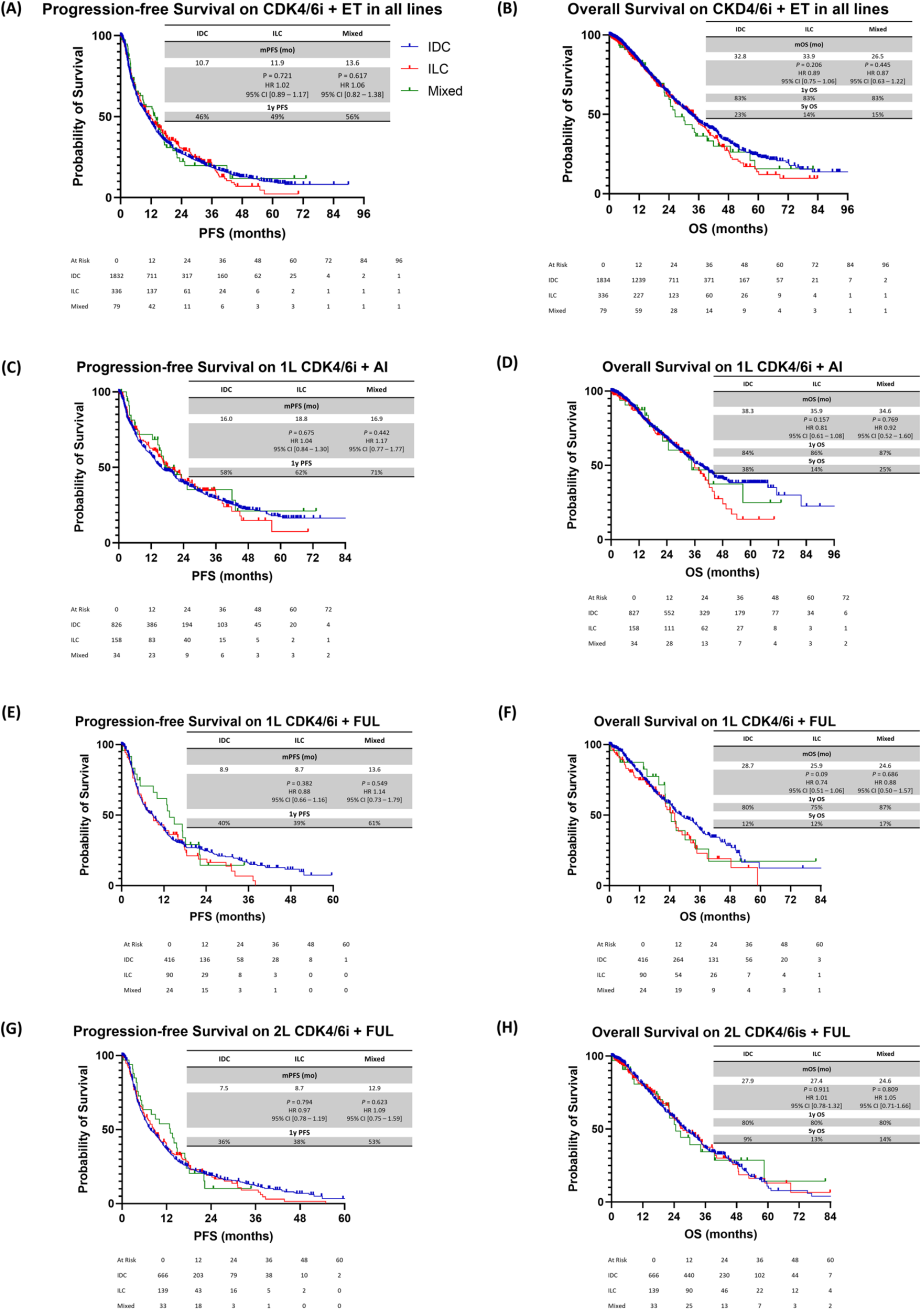
PFS and OS durations in HR+/HER2− mBC patients treated with CDK4/6is and ET. **A** PFS duration in all lines of therapy combined. **B** OS duration in all lines of therapy combined. **C** PFS duration on 1L CDK4/6is plus AI. **D** OS duration on 1L CDK4/6is plus AI. **E** PFS duration on 1L CDK4/6is plus FUL. **F** OS duration on 1L CDK4/6is plus FUL. **G** PFS duration on 2L+ CDK4/6is plus FUL. **H** OS duration on 2L+ CDK4/6is plus FUL. IDC invasive ductal carcinoma, ILC invasive lobular carcinoma, mixed mixed invasive ductal and lobular carcinoma, PFS progression-free survival, OS overall survival, CDK4/6is cyclin-dependent kinase 4 and 6 inhibitors, ET endocrine therapy, 1L, first line, 2L+ , second line and beyond, AI aromatase inhibitor, FUL fulvestrant, HR+, hormone receptor-positive, HER2− human epidermal growth factor receptor 2-negative.

Without stratifying by 1L or 2L+ therapy, we found no statistically significant difference in PFS and OS duration between IDC to ILC patients who received CDK4/6is plus ET (mPFS, 10.7 vs 11.9 months, hazard ratio (HR), 1.02; 95% confidence interval (CI), 0.89–1.17, *P* = 0.721; median OS (mOS) duration, 32.8 vs 33.9 months; HR, 0.89; 95% CI, 0.75–1.06; *P* = 0.206). Similar outcomes were seen when comparing IDC to mixed (Fig. 2A, B).

When CDK4/6is plus ET were given in the 1L setting, the difference in mPFS and mOS duration between histologies was not statistically significant (Fig. 1C, D). In patients who received 1L CDK4/6is plus AI, the mPFS duration was 16.0 months for IDC compared to 18.8 months for ILC (HR, 1.04; 95% CI, 0.84–1.30; *P* = 0.675) and 16.9 months for mixed (HR, 1.17; 95% CI, 0.77–1.77; *P* = 0.442) (Fig. 2C). The mOS duration was 38.3 months for IDC compared to 35.9 months for ILC (HR, 0.81; 95% CI, 0.61–1.08; *P* = 0.157) and 34.6 months for mixed (HR 0.92, 95% CI: 0.52–1.60, *P* = 0.769) (Fig. 2D). Similarly, when assessing patients who had been treated with CDK4/6is plus FUL in the 1L and 2L + setting, we found no statistically significant difference in mPFS and mOS durations among histologies (Fig. 2E–H).

Subgroup analysis in patients who received CDK4/6is plus ET showed no statistically significant differences in mPFS and mOS when stratified by race (Table 3). Analysis for mixed patients and patients who received everolimus or alpelisib were omitted due to the very small numbers of Hispanic and Black patients in those groups (Table 1). Similarly, “Others” were omitted from analysis given that they are not a homogenous group.

**Table 3:** Subgroup analysis based on race in patients treated with CDK4/6is plus ET.

	White		Black		Hispanic	
	IDC, *n* = 1375	ILC, *n* = 269	IDC, *n* = 165	ILC, *n* = 17	IDC, *n* = 147	ILC, *n* = 34
mPFS	10.7	11.9	11.7	7.5	8.9	10.1
	*P* = 0.125 HR 1.15 95% CI [0.96–1.35]		*P* = 0.309 HR 0.74 95% CI [0.39–1.40]		*P* = 0.813 HR 1.06 95% CI [0.63–1.77]	
mOS	30.8	26.5	28.1	16.0	24.3	35.4
	*P* = 0.115 HR 1.35 95% CI [0.85–1.36]		*P* = 0.160 HR 0.53 95% CI [0.24–1.16]		*P* = 0.194 HR 1.49 95% CI [0.86–2.88]	

*CDK4/6is* cyclin-dependent kinase 4 and 6 inhibitors, *ET* endocrine therapy, *IDC* invasive ductal carcinoma, *ILC* invasive lobular carcinoma, *mPFS* median progression-free survival, *mOS* median overall survival, *HR* hazard ratio, *CI* confidence interval.

We conducted multivariate analysis for the interaction of histology, race and ET backbone (AI versus FUL) with PFS and OS in patients treated with CDK4/6is plus ET (Tables 4 and 5). There was no statistically significant interaction observed.

**Table 4:** Multivariate Cox proportional hazards model to determine predictors of progression-free survival in patient treated with CDK4/6is pus ET.

Variables	MV-HR	95% CI	*P* value
Histology (compared to IDC)			
ILC	1.36	0.94–1.73	0.118
Mixed	0.86	0.46–1.45	0.604
Race (compared to White)			
Black	0.91	0.65–1.24	0.583
Hispanic	1.05	0.74–1.46	0.740
ET backbone (compared to AI)			
Fulvestrant	1.19	0.96–1.46	0.094

*CDK4/6is* cyclin-dependent kinase 4 and 6 inhibitors, *ET* endocrine therapy, *IDC* invasive ductal carcinoma, *ILC* invasive lobular carcinoma, *Mixed* mixed invasive ductal and lobular carcinoma, *AI* aromatase inhibitor, *MV-HR* multivariate hazard ratio, *CI* confidence interval.

**Table 5:** Multivariate Cox proportional hazards model to determine predictors of overall survival in patient treated with CDK4/6is plus ET.

Variables	MV-HR	95% CI	*P* value
Histology (compared to IDC)			
ILC	1.17	0.96–1.41	0.109
Mixed	0.98	0.62–1.47	0.949
Race (compared to White)			
Black	0.90	0.70–1.15	0.754
Hispanic	1.08	0.84–1.37	0.504
ET backbone (compared to AI)			
Fulvestrant	1.15	0.99–1.33	0.055

*CDK4/6is* cyclin-dependent kinase 4 and 6 inhibitors, *ET* endocrine therapy, *IDC* invasive ductal carcinoma, *ILC* invasive lobular carcinoma, *Mixed* mixed invasive ductal and lobular carcinoma, *AI* aromatase inhibitor, *MV-HR* multivariate hazard ratio, *CI* confidence interval.

**Fig. 3:**
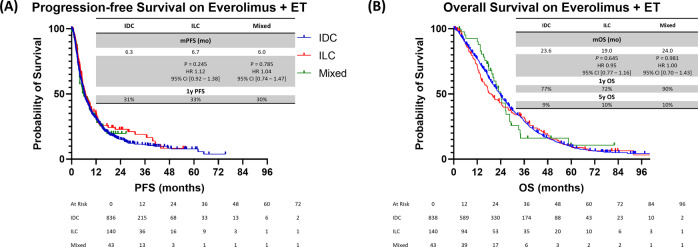
PFS and OS durations in HR+/HER2− mBC patients treated with the mTORi everolimus and ET. **A** PFS duration in 2L+. **B** OS duration in 2L+. IDC invasive ductal carcinoma, ILC invasive lobular carcinoma, mixed mixed invasive ductal and lobular carcinoma, PFS progression-free survival, OS overall survival, mTORi mammalian target of rapamycin inhibitor, ET endocrine therapy, 2L+ second line and beyond, HR+ hormone receptor-positive, HER2− human epidermal growth factor receptor 2-negative.

There were no statistically significant differences in PFS and OS duration between IDC and ILC patients who received everolimus plus ET (mPFS, 6.3 vs 6.7 months for IDC vs ILC; HR, 1.12; 95% CI, 0.92–1.38, *P* = 0.245; mOS duration, 23.6 vs 19.0 months for IDC vs ILC; HR, 0.95; 95% CI, 0.77–1.16; *P* = 0.645). Similar outcomes were seen when comparing IDC to mixed (Fig. 3).

**Fig. 4:**
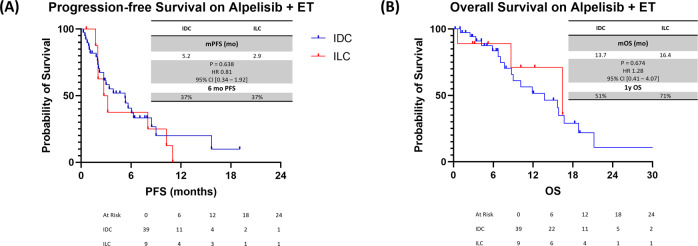
PFS and OS duration in HR + /HER2− mBC patients treated with PI3Ki alpelisib and ET. **A** PFS duration in 2L+. **B** OS duration in 2L+. IDC invasive ductal carcinoma, ILC invasive lobular carcinoma, PFS progression-free survival, OS overall survival, PI3Ki phosphoinositide 3-kinase inhibitor, ET endocrine therapy, 2L+ second line and beyond, HR+ hormone receptor-positive, HER2− human epidermal growth factor receptor 2-negative.

In addition, there were no statistically significant differences in PFS and OS duration between IDC and ILC patients who received alpelisib plus ET (mPFS duration, 5.2 vs 2.9 months for IDC vs ILC; HR, 0.81; 95% CI, 0.34–1.92; *P* = 0.638; mOS duration, 13.7 vs 16.4 months for IDC vs ILC; HR, 1.28; 95% CI, 0.41–4.07; *P* = 0.674) (Fig. 4).

## Discussion

To our knowledge, this is the first large retrospective study to compare histology-based outcomes of the addition of targeted therapies to ET in HR+/HER2− mBC patients. None of the previously reported studies that tested CDK4/6is, everolimus, or alpelisib highlighted differences in outcomes among IDC, ILC, or mixed histologies.

The only finding indicating that CDK4/6is are beneficial in ILC was reported by the U.S. Food and Drug Administration in a pooled analysis. In that study, both IDC and ILC patients benefited from the addition of a CDK4/6i to AI (PFS HR, 0.51 in IDC and 0.60 in ILC) and fulvestrant (PFS HRs, 0.52 and 0.43, respectively)^21^. Similarly, in an updated analysis, both IDC and ILC patients experienced a longer OS duration with the addition of a CDK4/6i to AI (HRs, 0.75 and 0.66, respectively)^22^. However, histologies were available for less than half of the patients. Similarly, our study showed that there was no difference in outcomes when patients with different histologies were treated with CDK4/6is + ET. Although there were no statistically significant differences in mOS duration among histologies in patients treated with CDK4/6is plus AI in the 1L setting, the 5-year OS rate was 2.5-fold higher in IDC than in ILC (5-year OS rates, 38% vs 14%, respectively, Fig. 2D). Similarly, although not statistically significant, Black patients with ILC treated with CDK4/6is plus ET had numerically worse mPFS (7.5 vs 11.7 months) and worse mOS (16.0 vs 28.1 months) when compared to those with IDC (Table 3). These observations are intriguing and hypothesis-generating.

One possible explanation for the aforementioned observation is that compared to IDC, ILC tends to have a higher frequency of de novo mutations that have been associated with CDK4/6is resistance, such as *PIK3CA*, *PTEN*, *AKT1*, and *FGFR1* mutations^23^. Furthermore, a hallmark of ILC is the loss of E-cadherin, which has been associated with the increased sensitivity of ILC cells to insulin-like growth factor 1; this, in turn, leads to phosphorylation and activation of the PTEN/PI3K/AKT signaling pathway, independent of oncogenic mutations in *PIK3CA*, *AKT1*, or *PTEN*^24,25^. AKT levels and activity are increased, which has been associated with CDK4/6i resistance^26^. Hypothetically, this difference is not observed in patients treated with 1L or 2L + FUL since IDC may acquire mutations that confer CDK4/6is resistance after disease progression on AI, resulting in a similar response to CDK4/6is in IDC and ILC. These results are hypothesis-generating and should be interpreted carefully. Further studies are warranted to better understand this difference in outcomes.

The predominant CDK4/6i used in our study was palbociclib (93% of patients who received CDK4/6is, Table 2); although cross-trial analysis is generally discouraged, the mPFS on 1L CDK4/6is + AI reported in our study is lower than that reported in the PALOMA-1 and -2 trials^27,28^. Our study reported a mPFS between 16.0 months (in IDC) and 18.8 months (in ILC) with patients treated with CDK4/6is + AI which is lower than that reported in the PALOMA-1 (20.2 months)^27^ and PALOMA-2 (24.8 months)^28^ trials. This discrepancy is consistent with other reported real-world data of palbociclib in combination with ET that showed similar shorter mPFS metrics when compared with more homogeneous prospective phase 3 clinical trial data^29,30^. One of the reasons for this discrepancy may be that the PALOMA-1 and -2 trials had patients from countries with much less pretreatment (i.e., more de novo cases and less prior exposure to chemotherapy and ET) compared to our population.

Patients with mixed histological type benefited in a similar fashion to those with IDC, regardless of the line of therapy and ET backbone used, consistent with the results of prior studies showing that the mixed histological type behaves more similarly to IDC than to ILC^3,31,32^.

ILC is associated with a higher rate of *PIK3CA* mutation than is IDC (~55% in ILC vs 35% in IDC)^23^. In the SOLAR-1 trial, the addition of alpelisib, a PI3K-α inhibitor, to ET significantly improved the mPFS duration of patients with *PIK3CA*-mutated mBC^33^. However, the study did not report outcomes based on histological type, and it is unknown whether ILC patients experienced the same benefit as the overall population. Although the number of patients who received alpelisib was small, our study showed that patients with ILC benefited similarly as patients with IDC (Fig. 4). However, these findings might be different if we had a larger sample given that pre-clinical studies have shown that ILC cells are very sensitive to PI3Kis and when E-cadherin loss is induced in IDC cell lines, these IDC cells became more sensitive to PI3Kis^34,35^.

ILCs tend to have constitutional activation of the PTEN/PI3K/AKT signaling pathway, resulting in increased sensitivity to insulin-like growth factor 1 receptor (IGF-1), PI3K, AKT, and MEK inhibitors in ILC models, but this has not been observed in IDC ones^34^. This led to the hypothesis that ILC patients would have improved outcomes when treated with everolimus, a drug that inhibits the downstream mTORC1 molecule of the PTEN/PI3K/AKT pathway. The BOLERO-2 trial evaluated the efficacy of everolimus in combination with exemestane in patients with HR+/HER2− mBC who experienced disease progression while undergoing ET^36^. A follow-up subgroup analysis showed that ILC patients experienced a greater benefit from the addition of everolimus to exemestane than from placebo plus exemestane (ORR, 14.1% vs. 0%; mPFS duration, 6.9 months vs. 4.2 months; HR, 0.59; 95% CI, 0.37–0.95)^37^. However, this study did not show whether ILC patients experienced a more pronounced benefit from the addition of everolimus than did IDC patients. In a study using pre-clinical ILC models, inhibition of mTOR signaling (using an mTORi) blocked the growth of ILC primary tumors as well as the progression of metastatic disease. However, primary tumors and distant metastases eventually acquired resistance after long-term treatment, despite continued effective suppression of mTOR signaling in cancer cells^35^. This can be one of the hypothetical reasons why our study did not show a superior mPFS in ILC patients compared to IDC.

The median PFS and OS durations reported here were consistent with those from previous studies performed using data from the same database^29^. The mPFS and mOS durations observed with 1L CDK4/6is plus FUL (Fig. 2E, F) were shorter than were those observed with 1L CDK4/6is plus AI (Fig. 2C, D), which can be explained by the fact that all patients treated with 1L CDK4/6is plus FUL experienced disease recurrence while on adjuvant AI.

Although the database in our study uses data that were prospectively collected, our study is still limited by the retrospective nature of our analysis. Moreover, 22% of patients were not included in the final analysis due to missing data. Finally, despite the ethnic diversity in the Houston area, our study under-represented key populations such as Hispanics (which represent 45% of the Houston population) and Black (which represent 22% of the Houston population).

In this large histology-based retrospective study, the addition of CDK4/6is, everolimus, or alpelisib to ET in patients with HR+/HER2− mBC led to a similar magnitude of benefit, irrespective of histology. These results are reassuring for patient with lobular and mixed histologies. Future studies are encouraged to be more inclusive by highlighting outcomes based on histology.

## Methods

### Study population and variables

Approval was obtained from the institutional review board at The University of Texas MD Anderson Cancer Center (MDACC, approval no. PA18-0386). A waiver of consent was obtained to ensure ethical standards of data use due to the retrospective nature of the study. In this study, we searched the prospectively collected data in the electronic BC database at The University of Texas MD Anderson Cancer Center (Houston, Texas) to identify patients with HR+/HER2− mBC who had been treated with ET in combination with targeted therapy (CDK4/6is, mTORi, or PI3Ki) between January 1, 2010, and December 31, 2021.

Data including patient demographics, treatment received, treatment duration, survival, and last follow-up were collected. Patients were first categorized based on the targeted therapy used (CDK4/6is, mTORi, or PI3Ki) in combination with ET, without stratifying by type of ET or line of therapy. Patients who received CDK4/6is plus ET were then categorized based on the line of therapy (first line [1L] versus the second line and beyond [2L+]) and the ET backbone (aromatase inhibitor [AI] versus fulvestrant [FUL]).

Progression-free survival (PFS) and overall survival (OS) duration data generated from all treatment categories were compared among all three histologies: IDC, ILC, and mixed.

### Statistical analysis

Wilcoxon rank-sum tests were used to compare the distribution of continuous variables among histological types. Fisher’s exact tests were used to compare the distribution of categorical variables.

The method of Kaplan and Meier was used to estimate the distribution of OS duration from the date of the initiation of treatment to the time of death or last follow-up. Patients who were still alive were censored at their last contact date. PFS duration was defined as the time from the date of the initiation of treatment to the date of the end of treatment. Patients who had no end date were censored at the time of last contact. Multivariate (MV) analysis using Cox regression was used to assess the association between co-variables and PFS/OS in patients treated with CDK4/6is + ET.

Survival distributions were compared among histologies using the log-rank test. All statistical analyses were performed using R software version 4.1.1 and a significance level of 5%. No adjustments were made for multiple testing. Figures were generated using GraphPad Prism 9.

## Data Availability

The data that support the findings of this study are available from the corresponding author, upon reasonable request.
